# Diagnostic value of positron emission tomography using amyloid-binding radiotracers for cardiac amyloidosis: a systematic review and meta-analysis

**DOI:** 10.1093/ehjimp/qyag141

**Published:** 2026-08-18

**Authors:** Amely Walser, Konstantinos Bitos, Andreas A Giannopoulos, Aju P Pazhenkottil, Valerie Treyer, Martin W Huellner, Ronny R Buechel, Philipp A Kaufmann, Dominik C Benz

**Affiliations:** Departments of Nuclear Medicine and Cardiology, University Hospital Zurich, Ramistrasse 100, Zurich 8091, Switzerland; Departments of Nuclear Medicine and Cardiology, University Hospital Zurich, Ramistrasse 100, Zurich 8091, Switzerland; Departments of Nuclear Medicine and Cardiology, University Hospital Zurich, Ramistrasse 100, Zurich 8091, Switzerland; Departments of Nuclear Medicine and Cardiology, University Hospital Zurich, Ramistrasse 100, Zurich 8091, Switzerland; Departments of Nuclear Medicine and Cardiology, University Hospital Zurich, Ramistrasse 100, Zurich 8091, Switzerland; Departments of Nuclear Medicine and Cardiology, University Hospital Zurich, Ramistrasse 100, Zurich 8091, Switzerland; Departments of Nuclear Medicine and Cardiology, University Hospital Zurich, Ramistrasse 100, Zurich 8091, Switzerland; Departments of Nuclear Medicine and Cardiology, University Hospital Zurich, Ramistrasse 100, Zurich 8091, Switzerland; Departments of Nuclear Medicine and Cardiology, University Hospital Zurich, Ramistrasse 100, Zurich 8091, Switzerland; Advanced Clinician Scientist Program, University Medicine Zurich (UMZH), University of Zurich, Zurich, Switzerland; Department of Cardiology, University Hospital Zurich, Zurich, Switzerland

**Keywords:** amyloidosis, cardiomyopathy, PET, diagnostic accuracy

## Abstract

**Aims:**

Amyloid-binding positron emission tomography (PET) tracers have emerged as a promising non-invasive tool for the diagnosis of cardiac amyloidosis (CA). This study aimed to assess the diagnostic accuracy of amyloid PET for detecting CA through a meta-analysis of diagnostic test accuracy.

**Methods and results:**

PubMed, Cochrane Library, Embase, and Web of Science were searched through 16 March 2026 for studies reporting the diagnostic performance of amyloid PET for CA. The primary analysis was a bivariate random-effects (Reitsma) model yielding summary sensitivity, specificity, the summary receiver operating characteristics curve with corresponding area under the curve (AUC), and positive and negative likelihood ratios. Subgroup analyses were performed by radiotracer and by amyloid subtype. Risk of bias was assessed using QUADAS-2 and publication bias by Deeks’ funnel-plot asymmetry test. Twenty studies comprising 577 patients (407 with CA, 170 controls) met inclusion criteria, covering five radiotracers (^11^C-PiB, ^18^F-florbetaben, ^18^F-florbetapir, ^18^F-flutemetamol, and ^124^I-evuzamitide). In the primary bivariate model (*k* = 17), amyloid PET demonstrated a summary sensitivity of 0.91 (95% CI 0.83–0.96), specificity of 0.90 (95% CI 0.79–0.96), and AUC of 0.96, with low between-study heterogeneity (sensitivity *I*^2^ = 13.4%; specificity *I*^2^ = 0%). Sensitivity was high and consistent across ^11^C-PiB, ^18^F-florbetaben, ^18^F-florbetapir, and ^124^I-evuzamitide (0.96–1.00), with the exception of ^18^F-flutemetamol, which showed lower and heterogeneous sensitivity (0.70).

**Conclusion:**

Amyloid PET demonstrates high sensitivity and specificity for CA detection, even though the evidence base remains limited in terms of the number and scope of available studies. Prospective multicentre studies in consecutive patients with suspected CA are required to establish its clinical role and standardize acquisition protocols.

## Graphical Abstract

**Graphical Abstract:**
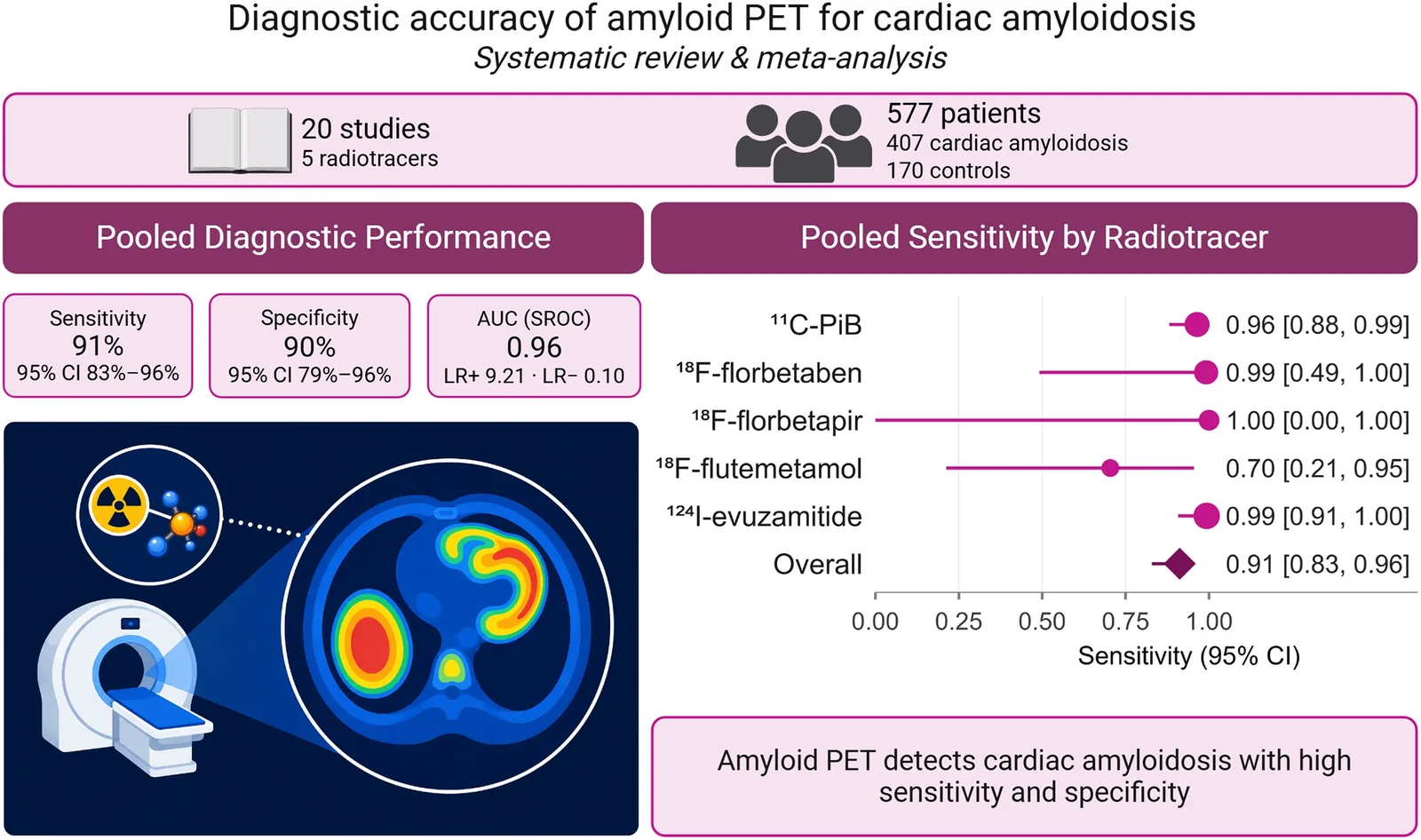

## Introduction

Cardiac amyloidosis (CA) is a progressive restrictive cardiomyopathy caused by the deposition of misfolded amyloid fibrils in the myocardium. The main forms are amyloid light-chain (AL) and transthyretin-related (ATTR) [wild-type (ATTRwt) or variant (ATTRv)] cardiomyopathy. Without treatment, the prognosis is poor: median survival from diagnosis is less than 1 year for AL-CA and 3–5 years for ATTR-CA.^1^ Fortunately, the therapeutic landscape has changed significantly over the past decade, and highly effective therapies are now available for both subtypes. This therapeutic progress has significantly increased the clinical need for an accurate and timely diagnosis.

Diagnostic evaluation for CA includes cardiac imaging, laboratory biomarkers, and, if necessary, histological confirmation via biopsy. Echocardiography and cardiac magnetic resonance (CMR) imaging can reveal morphological and functional abnormalities characteristic of CA, but neither modality achieves the specificity required for a definitive diagnosis. In ATTR-CA, nuclear medicine imaging with bone-avid radiotracers such as technetium-99m-labelled pyrophosphate (^99^ᵐTc-PYP), diphosphono-propandicarboxylic acid (DPD), or hydroxymethylenediphosphonate enables a non-invasive diagnosis. In the absence of a monoclonal protein, a grade 2 or 3 myocardial uptake on single-photon emission computed tomography (SPECT) has a specificity of nearly 100% for ATTR-CA, with no need for histological confirmation. However, sensitivity is limited to ∼72%, particularly in the very early stages of the disease or in cases of low cardiac uptake (Grade 1),^2^ and SPECT radiotracers are unsuitable for diagnosing AL and rare forms of CA. In systemic AL amyloidosis with cardiac involvement (AL-CA), diagnosis is based on the detection of a monoclonal protein combined with tissue confirmation through extracardiac (in the setting of typical morphological features of CA) or endomyocardial biopsy. Biopsy remains the gold standard for diagnosis in all subtypes when non-invasive tests do not provide clear results. However, it is invasive, and a focal myocardial biopsy does not provide information about the overall amyloid burden of the heart muscle.

Amyloid-specific positron emission tomography (PET) imaging has emerged as a promising non-invasive method for diagnosing CA. Several PET radiotracers, originally used in Alzheimer’s disease research to visualize cerebral β-amyloid plaques, have been subsequently applied to detect myocardial and systemic amyloid deposits.^3^ Unlike bone-avid SPECT tracers, amyloid PET tracers directly bind amyloid fibrils with high molecular affinity, enable quantification of amyloid burden in the myocardium, and have the potential to distinguish between AL and ATTR-CA. These include the thioflavin T derivatives ^11^C-Pittsburgh Compound B (^11^C-PiB) and ^18^F-flutemetamol, as well as the stilbene-based ^18^F-florbetapir and ^18^F-florbetaben. Additionally, ^124^I-evuzamitide, a novel broad-spectrum amyloid tracer, binds both amyloid fibrils and the glycosaminoglycans abundant on amyloid of any composition.^4,5^

Against this background, amyloid-specific PET imaging has gained increasing importance as a non-invasive, quantitative method capable of overcoming several of these diagnostic limitations. A growing number of studies have examined the diagnostic performance of various amyloid PET tracers in CA. This meta-analysis aims to provide a comprehensive and systematic evaluation of the diagnostic accuracy of amyloid PET imaging in CA, stratified by radiotracer and amyloid subtype.

## Methods

### Regulatory aspects

This systematic review and meta-analysis did not involve patients and therefore did not require ethical approval. Reporting follows the PRISMA 2020 statement^6^ and the PRISMA-DTA extension. The protocol was prospectively registered on PROSPERO (CRD420261343079).

### Search strategy and study selection

PubMed, Cochrane Library, Embase, and Web of Science were searched through 16 March 2026. The search combined terms related to CA with terms related to PET imaging and amyloid-binding radiotracers. The complete search strings, including MeSH terms where applicable, are provided in the Supplementary data online, *Table S1*.

Eligible studies were original clinical investigations evaluating amyloid-binding PET for the diagnosis of CA (AL or ATTR) reporting sensitivity, specificity, or 2 × 2 contingency counts [true positives (TP), false positives (FP), true negatives (TN), false negatives (FN)] in extractable form. A full list of extracted data items is provided in Supplementary data online, *Table S2*. Studies using ^18^F-sodium fluoride were excluded *a priori* because this tracer targets microcalcification rather than amyloid fibrils. Animal studies, reviews, case reports, and studies reporting only prognostic or therapy-monitoring outcomes were excluded. For overlapping cohorts from the same research group, the publication with the largest sample size or most complete reporting was retained (see Supplementary data online, *Table S3*). Two reviewers independently screened records and extracted data; disagreements were resolved by discussion.

### Data extraction and study quality

Search results were uploaded to Rayyan (www.rayyan.ai) for deduplication, title and abstract screening, full-text screening, and structured data extraction with blinded dual review. Extracted variables included study characteristics, amyloid subtype, PET protocol, reference standard, and diagnostic accuracy measures. Where TP, FP, TN, and FN were reported without sensitivity or specificity, these were calculated; where 95% confidence intervals were missing or based on methods other than Clopper–Pearson, intervals were uniformly recomputed using the Clopper–Pearson exact method to ensure comparability across studies.

Methodological quality was assessed using the Quality Assessment of Diagnostic Accuracy Studies 2 (QUADAS-2) tool^7^ across four domains: patient selection, index test, reference standard, and flow and timing.

### Data synthesis and analysis

The primary analysis was a bivariate random-effects model (Reitsma model^8^) yielding summary sensitivity and specificity, the summary receiver operating characteristic (SROC) curve and corresponding AUC, and the positive likelihood ratio (LR+), negative likelihood ratio (LR−), and diagnostic odds ratio (DOR); studies with complete 2 × 2 contingency tables contributed to this analysis. Univariate random-effects pooling of sensitivity and specificity (logit transformation, Clopper–Pearson individual-study CIs) was performed in parallel and is displayed as coupled forest plots. Studies with only sensitivity or only specificity reconstructable contributed to the corresponding univariate pool; studies of diagnostic relevance without extractable binary data were retained for qualitative synthesis. Because the majority of primary-model studies reported zero false-positive observations, the SROC was extrapolated across [0,1] for visualization, and 95% confidence and prediction regions of the summary operating point were displayed per the Cochrane DTA Handbook.

Subgroup analyses of sensitivity and specificity were performed by radiotracer and by amyloid subtype. For the subtype analysis, mixed cohorts were split into AL and ATTR sensitivity arms so that each study contributed to both subgroups. Specificity was not stratified, as control groups were non-amyloid. Quantitative myocardial uptake was compared between AL and ATTR in the subset of β-amyloid studies that reported a continuous myocardial retention metric separately by subtype. The standardized mean difference (Hedges’ g, corrected for small-sample bias) was pooled using a random-effects model (restricted maximum-likelihood estimator), with 95% confidence and prediction intervals. Where a study reported more than one metric, the retention index was used to maximize comparability. For one study reporting myocardial tracer retention (%) rather than a retention index, this analogous retention metric was used. For studies reporting median and range or interquartile range, mean and standard deviation were estimated using the method of Wan *et al*.^9^ A sensitivity analysis restricted to retention-index studies was performed.

### Heterogeneity and publication bias

Heterogeneity was estimated using the *I*^2^ statistic, presenting the percentage of variability between studies due to heterogeneity rather than chance.^10^ A meta-regression to explore its sources was planned *a priori* and was to be performed if substantial heterogeneity was present (*I*^2^ > 50%).^11^ Publication bias was assessed by visual inspection of the funnel plot and formally tested using Deeks’ funnel-plot asymmetry test, with *P* < 0.10 considered indicative of significant asymmetry.^12^ A continuity correction of 0.5 was applied for zero-cell counts. Two-sided *P* ≤ 0.05 was considered statistically significant. Analyses were performed in R (version 4.5.1) using the packages mada (version 0.5.12) and meta (version 8.2.1).

## Results

### Literature search and study selection

**Figure 1:**
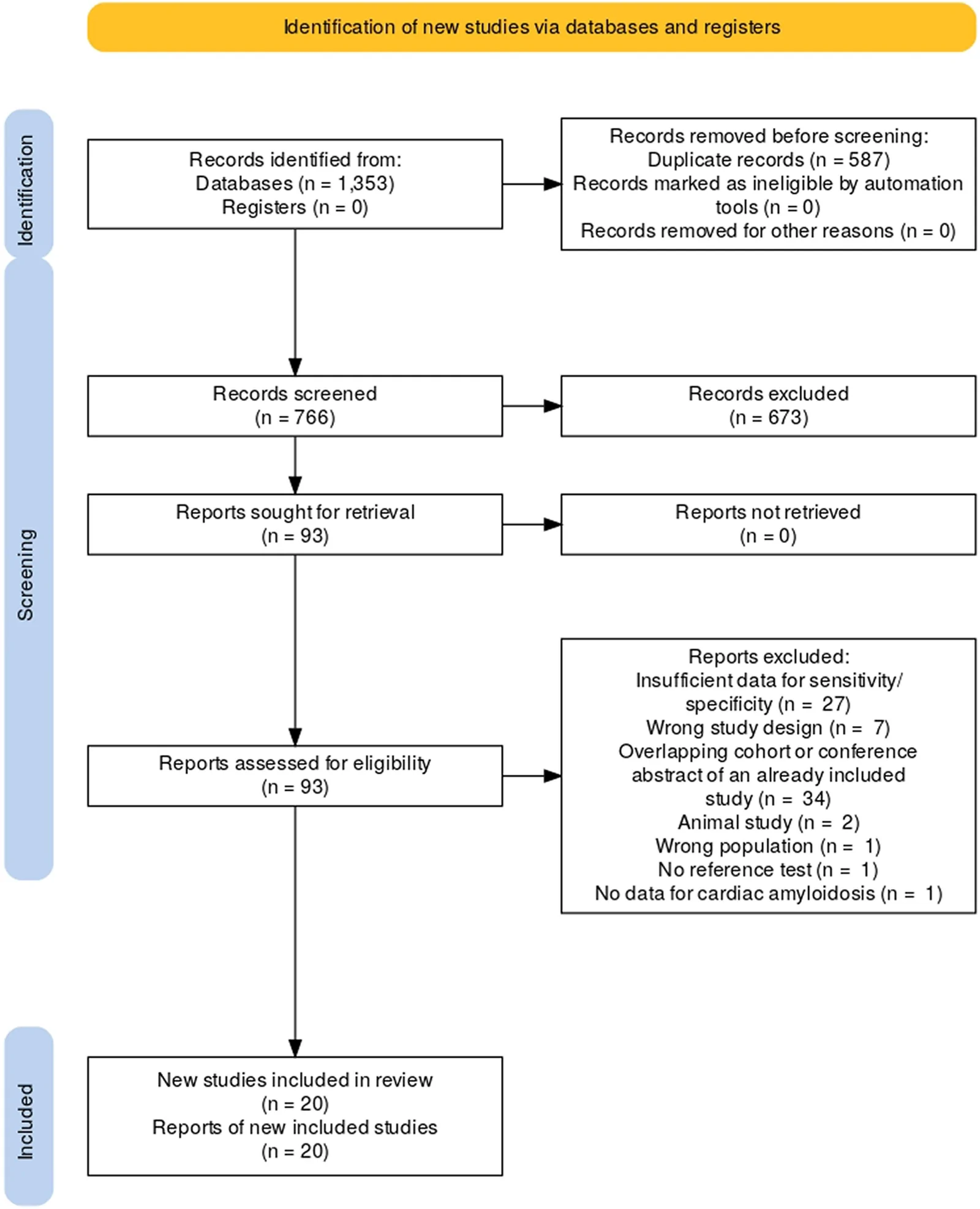
PRISMA flow diagram of study selection.

The search identified 1353 records. After removal of 587 duplicates, 766 records were screened based on title and abstract, of which 673 were excluded. Subsequently, 93 full-text articles were assessed for eligibility, of which 73 were excluded: 34 for being abstracts of an already included study or for cohort overlap with another study from the same research group, 27 for insufficient 2 × 2 data, 7 for ineligible study design, 2 for being animal studies, and 3 for other reasons (*Figure 1*). Twenty studies met the inclusion criteria.

### Quality assessment

The summary of concerns regarding potential for bias and applicability, based on the modified QUADAS-2, is presented in Supplementary data online, *Figure S1*. Overall, the quality of the included studies is rated as satisfactory. The most frequent concern was patient selection, reflecting the predominantly case-control design of included studies.

### Study characteristics

The 20 included studies were published between 2013 and 2025 and comprised 15 prospective and 5 retrospective cohorts from 11 countries. Recruitment was predominantly case-control, comparing patients with confirmed CA against separately enrolled control groups rather than consecutive series of patients with suspected disease.

Across all 20 studies, the tracer distribution was ^11^C-PiB (*n* = 7), ^18^F-florbetaben (*n* = 4), ^18^F-florbetapir (*n* = 3), ^18^F-flutemetamol (*n* = 3), and ^124^I-evuzamitide (*n* = 3), comprising 407 patients with CA and 170 non-amyloid controls (577 total). Seventeen studies with complete 2 × 2 tables contributed to the primary bivariate analysis and the univariate specificity pool; all 20 contributed to the univariate sensitivity pool. Three studies^13–15^ were restricted to the sensitivity pool, having either no controls^14^ or control groups composed exclusively of AL amyloidosis patients without conventional cardiac involvement.^13,15^

With respect to amyloid subtype, six studies included AL only, one included ATTR only [ATTRv (V30M)], and 12 included mixed AL/ATTR populations; typing was incomplete in one study. Endomyocardial biopsy served as the sole reference standard in one study,^16^ while the remaining 19 combined extracardiac biopsy with imaging and clinical criteria. Study and PET characteristics are summarized in *Tables 1* and *2*. Additional studies relevant to amyloid PET but not eligible for quantitative synthesis are summarized in *Table 3*. Most studies used PET/CT, with PET/MR in three and a mixed protocol in one. Administered activity and acquisition timing varied considerably between studies, and positivity thresholds were heterogeneous, several of them derived from in-sample receiver operating characteristics (ROC) optimization.

**Table 1:** Characteristics of the included studies

Study	Country	Design	CA subtype	N CA	N Ctrl	Control type	Age (year)	Reference standard
^11^C-PiB								
Antoni (2013)^17^	Sweden	P	Mixed	10	5	HC	66	Adipose biopsy + echo + EMB (subset)
Lee (2015)^16^	South Korea	P	AL	15	7	Disease (MGUS, biopsy-neg.)	64	EMB + Congo red + IHC
Minamimoto (2018)^18^	Japan	P	AL	3	6	Disease (suspected CA, ruled out)	64	EMB + clinical follow-up
Ezawa (2018)^19^	Japan	P	Mixed	12	4	Mixed (3 HC + 1 TTR carrier)	23–82	Clinical + LV wall thickness >12 mm
Rosengren (2020)^20^	Sweden/Denmark	P	Mixed (15 AL + 21 ATTR)	36	15	Mixed (7 disease + 8 HC)	72	EMB or fat-pad biopsy + echo (Gertz criteria)
Bi (2022)^21^	China	R	Unknown	13	18	Mixed (10 disease + 8 HC)	62.9	Extracardiac biopsy + CMR + clinical
Wang (2022)^15^	China	R	AL	13	7	Mixed (7 pAL non-CA + 3 HC)	59.5	BCSH/ISA + tissue biopsy + Congo red + IF
^18^F-florbetaben								
Law (2016)^22^	Australia	P	Mixed	10	4	Disease (hypertensive HD)	NR	EMB (ATTR) or extracardiac biopsy (AL) + ISA criteria
Kircher (2019)^23^	Germany	R	Mixed (8 AL, 5 ATTR, 1 AA)	14	8	Disease (LCDD, MM, CAD, FMF, etc.)	65	EMB or extracardiac biopsy + clinical/echo/scintigraphy
Genovesi (2021)^24^	Italy	P	Mixed (20 AL + 20 ATTR)	40	20	Disease (HCM, hypertensive HD, DCM)	73	Biopsy + clinical/biomarker + echo + DPD scintigraphy
Milani (2024)^25^	Italy	P	AL	24	3	Disease (within-cohort ischaemic HD)	NR	Biopsy + NT-proBNP
^18^F-florbetapir								
Dorbala (2014)^26^	USA	P	Mixed	9	5	Mixed (3 HC + 2 non-ischaemic HF)	64	EMB or extracardiac biopsy + echo
Manwani (2018)^14^	UK	P	AL	15	0	None (single-arm)	58	Tissue biopsy + ISA criteria (no EMB required)
Cuddy (2020)^13^	USA	P	AL	25	10	AL non-CA (preclinical)	62	Biopsy + amyloid typing (Gertz criteria)
^18^F-flutemetamol								
Dietemann (2019)^27^	Switzerland	R	Mixed (8 ATTR + 1 AL)	9	3	Disease (suspected CA, ruled out)	NR	Extracardiac biopsy + scintigraphy + CMR (no EMB)
Möckelind (2020)^28^	Sweden	P	ATTR (ATTRv)	17	6	Mixed (5 Ad + 1 HC)	61	Fat pad biopsy (ATTRv (V30M)) + clinical
Papathanasiou (2022)^29^	Germany	R	Mixed	12	5	Disease (non-amyloid HF)	71.3	EMB (*n* = 9), or scintigraphy + AL workup, or variant ATTR + CMR
^124^I-evuzamitide								
Wall (2023)^30^	USA	P	Mixed	26	7	Mixed (5 HC + 2 TTR carriers)	65.5	Biopsy + scintigraphy + clinical
Clerc (2023)^31^	USA	P	Mixed	24	20	HC	74	Biopsy + clinical
Masri (2025)^32^	USA	P	Mixed (57 ATTR + 20 AL + 3 ApoA1/A4)	80	17	Disease (non-amyloid)	NR	NR

AA, serum amyloid A amyloidosis; Ad, Alzheimer’s disease; AL, immunoglobulin light chain amyloidosis; ApoA1/A4, apolipoprotein A-I/A-IV amyloidosis; ATTR, transthyretin amyloidosis; ATTRv, variant transthyretin amyloidosis; ATTRwt, wild-type transthyretin amyloidosis; BCSH, British Committee for Standards in Haematology; CA, cardiac amyloidosis; CAD, coronary artery disease; CMR, cardiac magnetic resonance; DCM, dilated cardiomyopathy; DPD, 3,3-diphosphono-1,2-propanodicarboxylic acid; EMB, endomyocardial biopsy; FMF, familial Mediterranean fever; HC, healthy controls; HCM, hypertrophic cardiomyopathy; HD, heart disease; HF, heart failure; IF, immunofixation; IHC, immunohistochemistry; ISA, International Society of Amyloidosis; LCDD, light chain deposition disease; MGUS, monoclonal gammopathy of undetermined significance; MM, multiple myeloma; NR, not reported; NT-proBNP, N-terminal pro-B-type natriuretic peptide; P, prospective; R, retrospective.

**Table 2:** PET characteristics of the included studies

Study	Tracer	Modality	Tracer dose (MBq)	Acquisition (min)	Reading method	Quantitative parameters	Cut-off
Antoni (2013)^17^	^11^C-PiB	PET/CT	6 MBq/kg (∼420 MBq for 70 kg)	D: 0–25 min; S: 15–25 min p.i.	Both	RI, MBF	Visual positive
Lee (2015)^16^	^11^C-PiB	PET/CT	555 MBq	S: 30–33 min p.i.	Both	SUV ratio (LV/aorta)	SUV ratio ≥ 2.0
Minamimoto (2018)^18^	^11^C-PiB	PET/CT	620 ± 168 MBq (range 366–746)	S: 40–60 min p.i.	Both	SUVmax, SUVmean, M/B ratio	Visual positive
Ezawa (2018)^19^	^11^C-PiB	PET/CT	568 MBq	S: 30–44 min p.i.	Visual	Visual grading (+/++/+++)	Visual positive (grade ≥+)
Rosengren (2020)^20^	^11^C-PiB	PET/CT	5 MBq/kg	D: 0–35 min; S: 10–20 min p.i.	Both	SUVR, RI	SUVR > 1.09; RI > 0.037 min^−1^
Bi (2022)^21^	^11^C-PiB	PET/MR	555 MBq	S: 30–50 min p.i.	Both	SUVmax, SUVmean, TBRmax	TBRmax > 1.09
Wang (2022)^15^	^11^C-PiB	PET/MR	NR	S: Whole-body, immediate post-injection	Both	SUVmax (cardiac)	SUVmax > 0 (cardiac)
Law (2016)^22^	^18^F-florbetaben	PET/CT	4 MBq/kg (∼259 ± 41 MBq)	D: 0–80 min; S: 15–75 min p.i.	Both	SUVmean, TBR, RI, %MR	%MR > 40%
Kircher (2019)^23^	^18^F-florbetaben	PET/CT	313 ± 26 MBq (range 260–375)	D: 0–30 min; S: 10–30 min p.i.	Both	SUVmean, MTR%	MTR ≤ 36%
Genovesi (2021)^24^	^18^F-florbetaben	PET/CT	300 MBq	D: 0–60 min; S: 5–110 min p.i. (multi-TP)	Both	SUVmean, H/Bkg ratio, MV, RI	Visual (late uptake pattern)
Milani (2024)^25^	^18^F-florbetaben	PET/CT	NR	D: 0–50 min; S: 15/50/110 min p.i. (multi-TP)	Quantitative	MTR at 15/50/110 min	Delayed uptake pattern
Dorbala (2014)^26^	^18^F-florbetapir	PET/CT	222 MBq	D: 0–60 min; S: 10–30 min p.i.	Both	RI, TBR, SUVmean, LV/liver SUV ratio	RI > 0.025 min^−1^ and TBR > 1.45
Manwani (2018)^14^	^18^F-florbetapir	PET/CT	331 MBq (range 294–370)	D: 0–60 min; S: 10–30 min p.i.	Both	LV RI, SUVmax, SUVmean	Visual positive (no formal cut-off)
Cuddy (2020)^13^	^18^F-florbetapir	PET/CT	355 ± 48 MBq	D: 0–60 min; S: 10–30 min p.i.	Both	RI	RI > 0.03 min^−1^
Dietemann (2019)^27^	^18^F-flutemetamol	PET/CT	360 MBq	D: 0–30 min; S: 10–30 min p.i.	Both	SUVmean, TBR	Visual positive (no formal cut-off)
Möckelind (2020)^28^	^18^F-flutemetamol	PET/CT	184 MBq	D: 0–60 min; S: 30–36 min p.i.	Both	IVS SUVmean, SUVmax, RI, amyloid burden	IVS SUV > 1.46 at 30 min
Papathanasiou (2022)^29^	^18^F-flutemetamol	Mixed (PET/MR *n* = 13, PET/CT *n* = 4)	182.1 ± 18.5 MBq	D: 0–30 min (subset *n* = 5); S: ∼78 min p.i.	Both	SUVmax, SUVmean, TBRmax, TBRmean, RI (subset *n* = 5)	Visual positive
Wall (2023)^30^	^124^I-evuzamitide	PET/CT	71.5 MBq	S: ∼5.2 h p.i.	Both	Visual + semiquant.	Visual positive
Clerc (2023)^31^	^124^I-evuzamitide	PET/CT	37 MBq	S: 5 h p.i. (30 min duration)	Quantitative	LV %ID, SUVmean, SUVmax, CAA, TBR	%ID > 0.38 (Youden)
Masri (2025)^32^	^124^I-evuzamitide	PET/MR	39 MBq	NR	Quantitative	Mean LV SUVR	Mean SUVR > 1.31 (Youden)

%ID, percentage of injected dose; %MR, percentage myocardial retention; CAA, cardiac amyloid activity; D, dynamic acquisition; H/Bkg, heart-to-background ratio; IVS, interventricular septum; M/B, myocardium-to-blood ratio; MBF, myocardial blood flow; MBq, megabecquerel; MTR, myocardial tracer retention; MV, molecular volume; NR, not reported; PET/CT, positron emission tomography/computed tomography; PET/MR, positron emission tomography/magnetic resonance; p.i., post-injection; RI, retention index; S, static acquisition; SUV, standardized uptake value; SUVR, standardized uptake value ratio; TBR, target-to-background ratio.

**Table 3:** Studies relevant to amyloid PET in cardiac amyloidosis but excluded from quantitative synthesis

Study (year)^Ref^	Population	Key finding/clinical implication	Reason not pooled
^11^C-PiB			
Hong (2025)^41^	AL/ATTR (NR)	Sequential ^11^C-PiB + ^99^ᵐTc-DPD improves AL/ATTR subtyping	Combined modality (PET + SPECT); standalone PET not isolatable
Takasone (2020)^40^	30 AL/ATTR, 17 HC	SUVmax differs by ATTR subtype (with PYP)	Combined modality
Pilebro (2018)^38^	10 ATTRv, 5 HC	Myocardial RI elevated in all ATTRv, incl. DPD-negative	Mechanistic fibril-type study; no diagnostic 2 × 2
Choi (2022)^39^	58 AL	^11^C-PiB uptake independently predicts outcome in AL-CA	Prognostic outcome only; no diagnostic 2 × 2 data
^18^F-florbetaben			
Santarelli (2021)^42^	15 AL, 13 ATTR, 19 HC	Deep-learning classifier separates AL/ATTR on early images	Non-standard analysis; no binary cut-off
Santarelli (2022)^43^	11 AL, 10 ATTR, 15 HC	Kinetic modelling distinguishes controls, AL and ATTR	Non-standard analysis; no binary cut-off
Sprauel (2026)^47^	AL/ATTR (NR)	Detects early multi-organ involvement before structural change	Data not in extractable 2 × 2 format
D’Estanque (2017)^45^	1 AL	Whole-body uptake in heart and other organs	Single-case report; no diagnostic 2 × 2
Baratto (2018)^44^	7 AL, 2 HC	Whole-body ^18^F-florbetaben localizes systemic AL	Whole-body focus; cardiac 2 × 2 not isolatable
Seo (2019)^46^	6 AL	^18^F-florbetaben accurately detects systemic amyloid	Systemic detection only; no controls, cardiac 2 × 2 not isolatable
^18^F-florbetapir			
Osborne (2015)^48^	8 CA, 3 HC	SUV kinetics separate CA from controls	SUV kinetics only; no binary cut-off
Mestre-Torres (2018)^49^	3 ATTR, 22 HC	No cardiac uptake in any ATTR patient	Sensitivity not estimable (0 true positives)
^124^I-evuzamitide			
Smiley (2025)^52^	25 ATTRwt/v	Detects ATTR when ^99^ᵐTc-PYP negative	Insufficient 2 × 2 data (preclinical readout)

AL, immunoglobulin light chain amyloidosis; ATTR, transthyretin amyloidosis (v, variant; wt, wild-type); CA, cardiac amyloidosis; DPD, 3,3-diphosphono-1,2-propanodicarboxylic acid; FBB, florbetaben; HC, healthy controls; NR, not reported; PYP, pyrophosphate; RI, retention index; SUV(R), standardized uptake value (ratio); 2 × 2, 2 × 2 contingency table.

### Diagnostic performance

**Figure 2:**
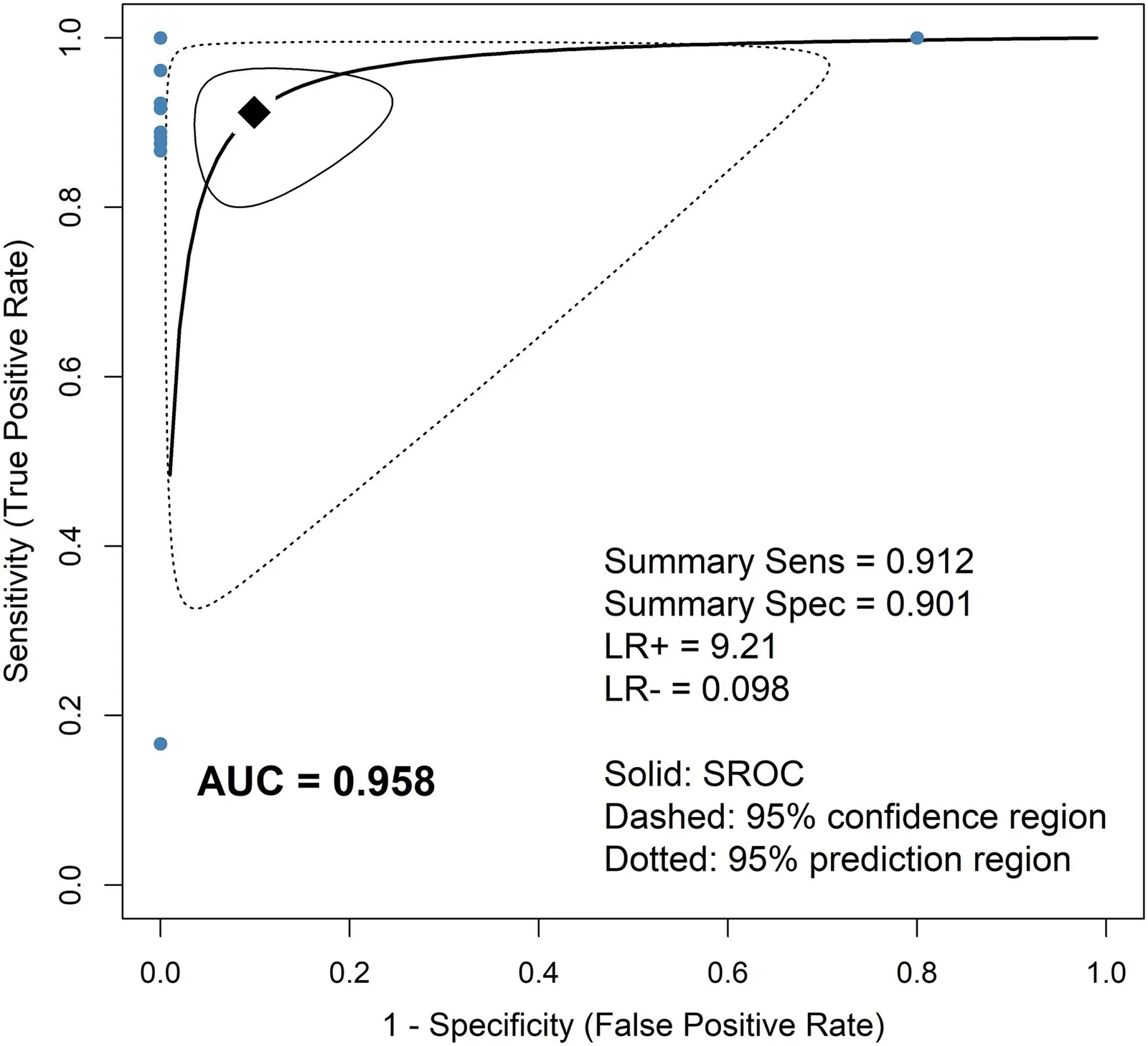
Summary receiver operating characteristic (SROC) curve of amyloid PET for the detection of cardiac amyloidosis. Bivariate Reitsma model (*k* = 17). The filled diamond marks the summary operating point (sensitivity 0.91; specificity 0.90); inner solid line, 95% confidence region; outer dotted line, 95% prediction region. The curve was extrapolated across [0,1] for visualization, as 16 of 17 studies reported no false positives. AUC, area under the curve.

In the primary bivariate Reitsma model (*k* = 17), amyloid PET showed a summary sensitivity of 0.91 (95% CI 0.83–0.96) and a summary specificity of 0.90 (95% CI 0.79–0.96). The positive and negative likelihood ratios were 9.21 and 0.10, the diagnostic odds ratio was 94.4, and the area under the SROC curve was 0.96 (*Figure 2*). The summary operating point is shown together with its 95% confidence and prediction regions. Because 16 of 17 studies reported zero false-positive observations, the SROC curve was extrapolated across the full [0,1] range for visualization.

**Figure 3:**
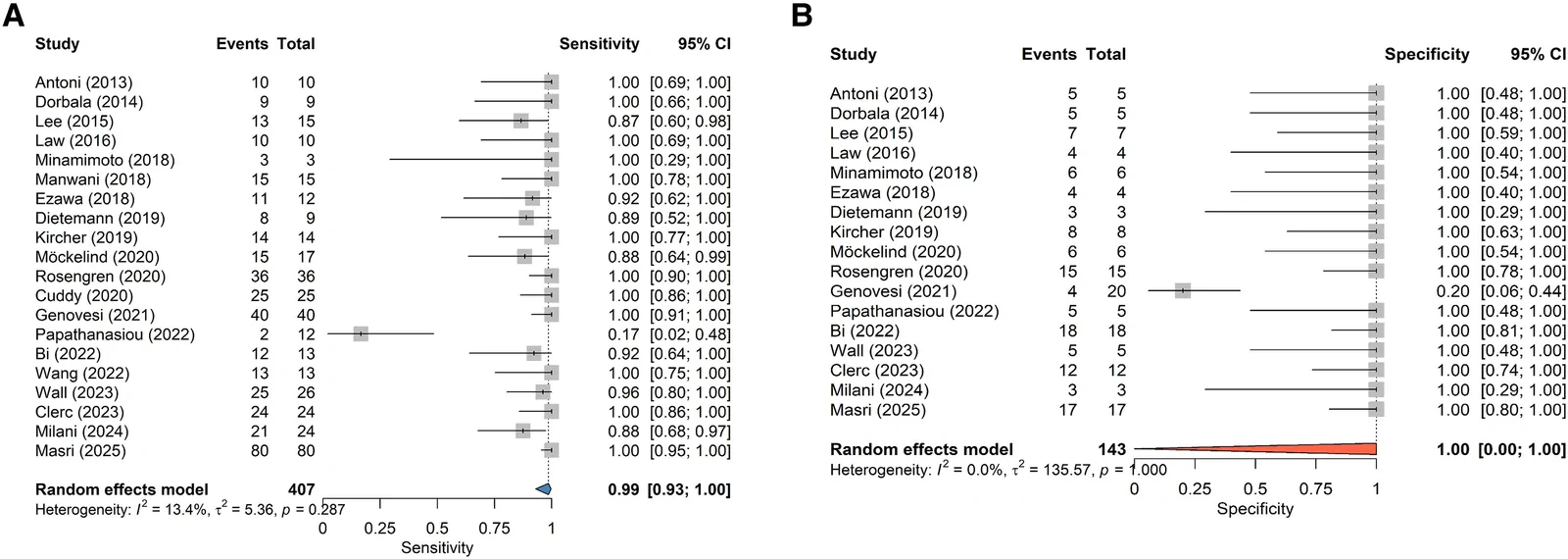
Coupled forest plots of sensitivity and specificity of amyloid PET for the detection of cardiac amyloidosis. Sensitivity (*A*, *k* = 20) and specificity (*B*, *k* = 17) from univariate random-effects models (logit transformation); individual-study confidence intervals by the Clopper–Pearson method. Diamonds represent pooled estimates. CI, confidence interval; *I*^2^, heterogeneity statistic.

Univariate random-effects pooling across all 20 studies yielded a summary sensitivity of 0.99 (95% CI 0.93–1.00). The univariate specificity estimate was uninformative owing to a ceiling effect, as 16 of 17 studies reported a specificity of 1.00 against non-amyloid controls. The bivariate estimate, therefore, serves as the summary specificity throughout. Coupled forest plots of the univariate analyses are shown in *Figure 3*. Univariate random-effects pooling yielded a diagnostic odds ratio of 119.96 (95% CI 50.50–285.00) with negligible between-study heterogeneity (see Supplementary data online, *Figure S2*).

### Subgroup analyses

**Figure 4:**
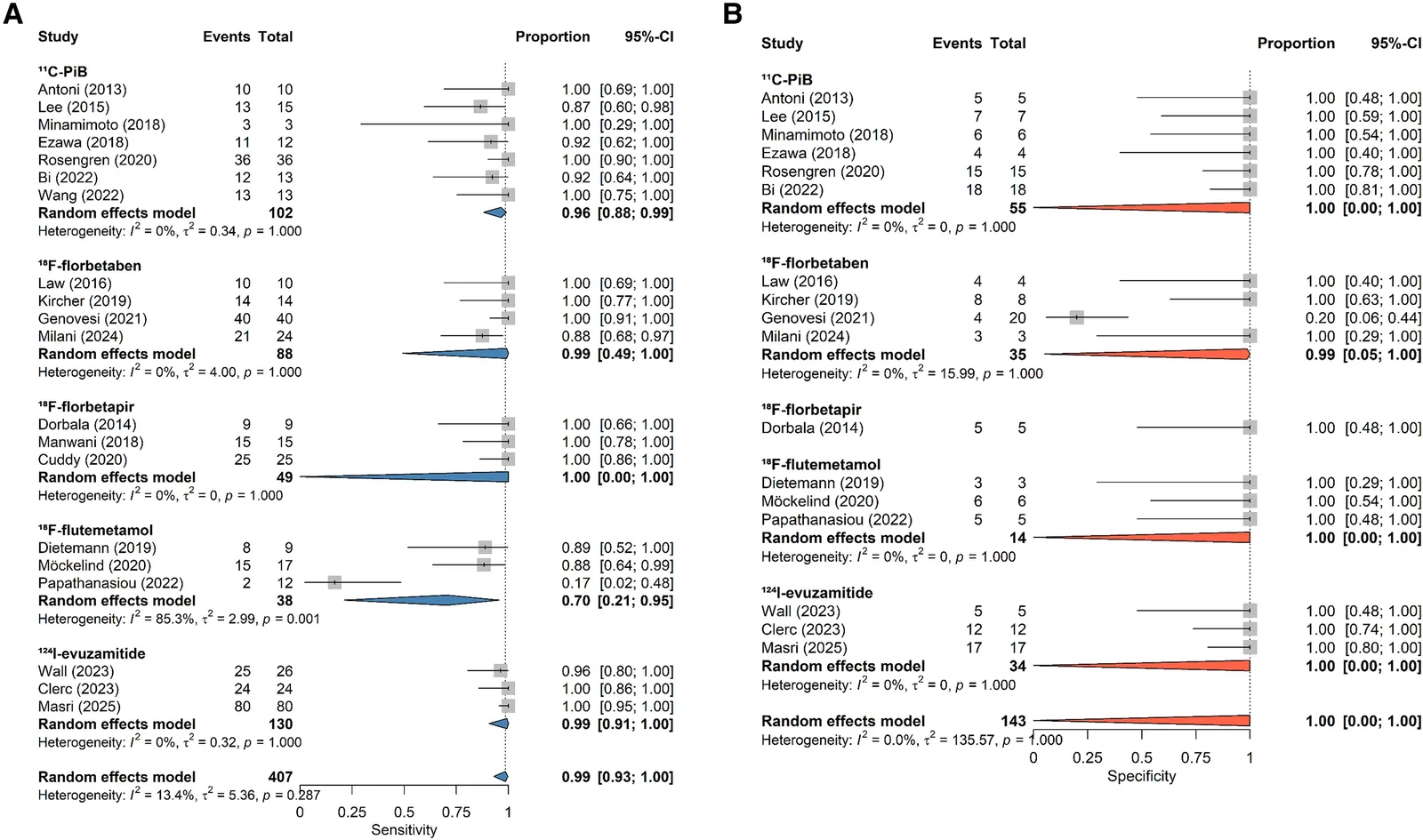
Subgroup forest plots of sensitivity and specificity stratified by radiotracer type. Sensitivity (*A*, *k* = 20) and specificity (*B*, *k* = 17) stratified by radiotracer from univariate random-effects models. Test for subgroup differences in sensitivity, *P* = 0.17. The univariate specificity pool reaches a ceiling (most studies 1.00); the bivariate model provides the summary specificity reported in the text. *I*^2^, within-subgroup heterogeneity statistic.

**By radiotracer (*Figure 4*).** The test for subgroup differences in sensitivity was not significant (*P* = 0.17). Pooled sensitivity was high and consistent across ^11^C-PiB, ^18^F-florbetaben, ^18^F-florbetapir, and ^124^I-evuzamitide (0.96–1.00), with specificity at or near 1.00 wherever non-amyloid controls allowed its estimation. The exception was ^18^F-flutemetamol (*k* = 3), which showed the lowest and most heterogeneous sensitivity (0.70, 95% CI 0.21–0.95, *I*^2^ = 85.3%).

**Figure 5:**
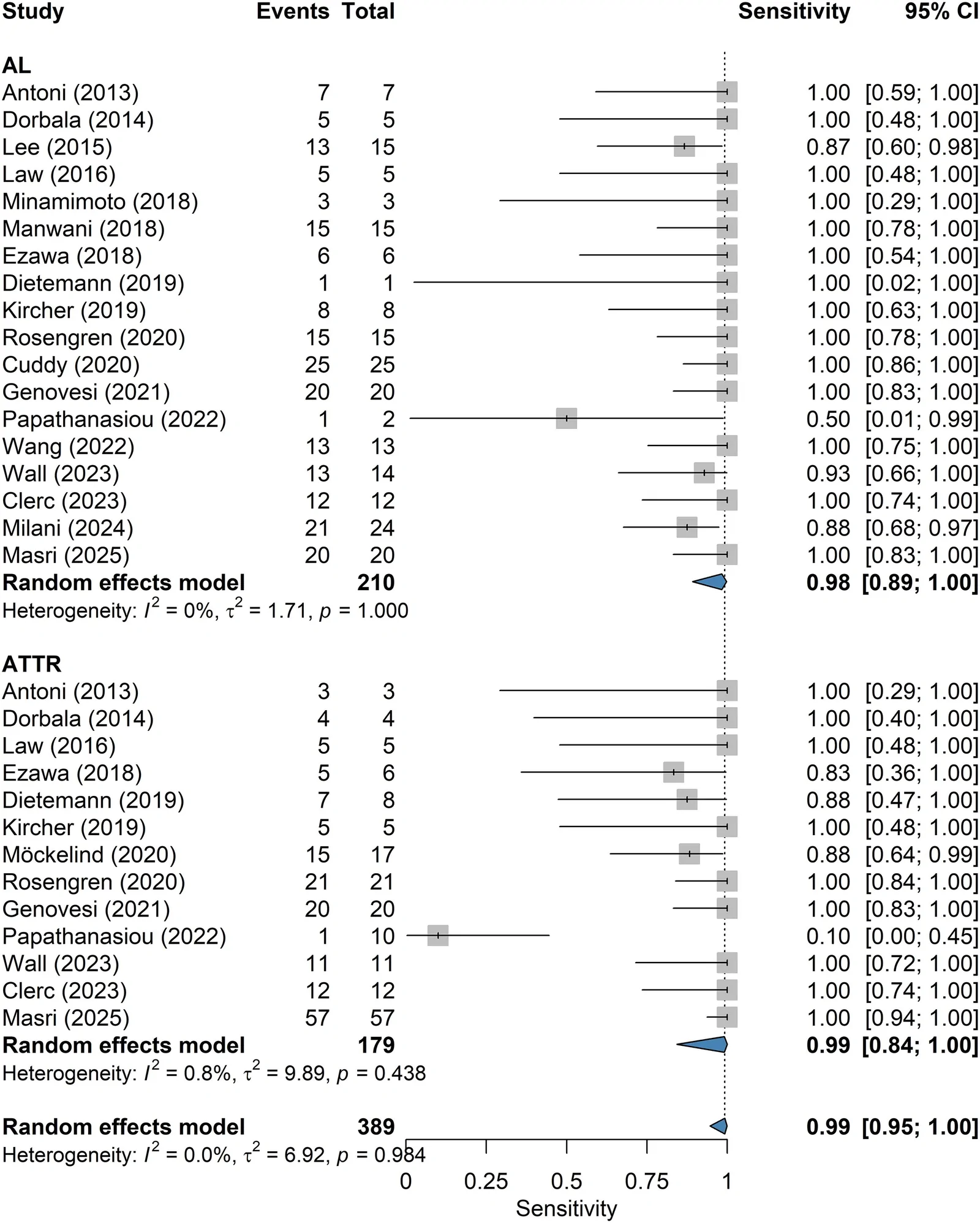
Arm-level sensitivity of amyloid PET for cardiac amyloidosis stratified by amyloid subtype. Mixed cohorts were split into AL and ATTR sensitivity arms, so that a study reporting both subtypes contributes to both subgroups. Pooled by univariate random-effects model (logit transformation); individual-study confidence intervals by the Clopper–Pearson method. Sensitivity was comparable between subtypes (AL, *k* = 18, 0.98; ATTR, *k* = 13, 0.99; subgroup difference χ^2^_1_ = 0.11, *P* = 0.74). AL, light-chain amyloidosis; ATTR, transthyretin amyloidosis; CI, confidence interval; *I*^2^, heterogeneity statistic.

**By amyloid subtype**. As most cohorts were mixed, each study was split into AL and ATTR sensitivity arms. Arm-level sensitivity was high and comparable between subtypes (AL: *k* = 18, 0.98; ATTR: *k* = 13, 0.99; subgroup difference *P* = 0.74; *Figure 5*).

**Figure 6:**
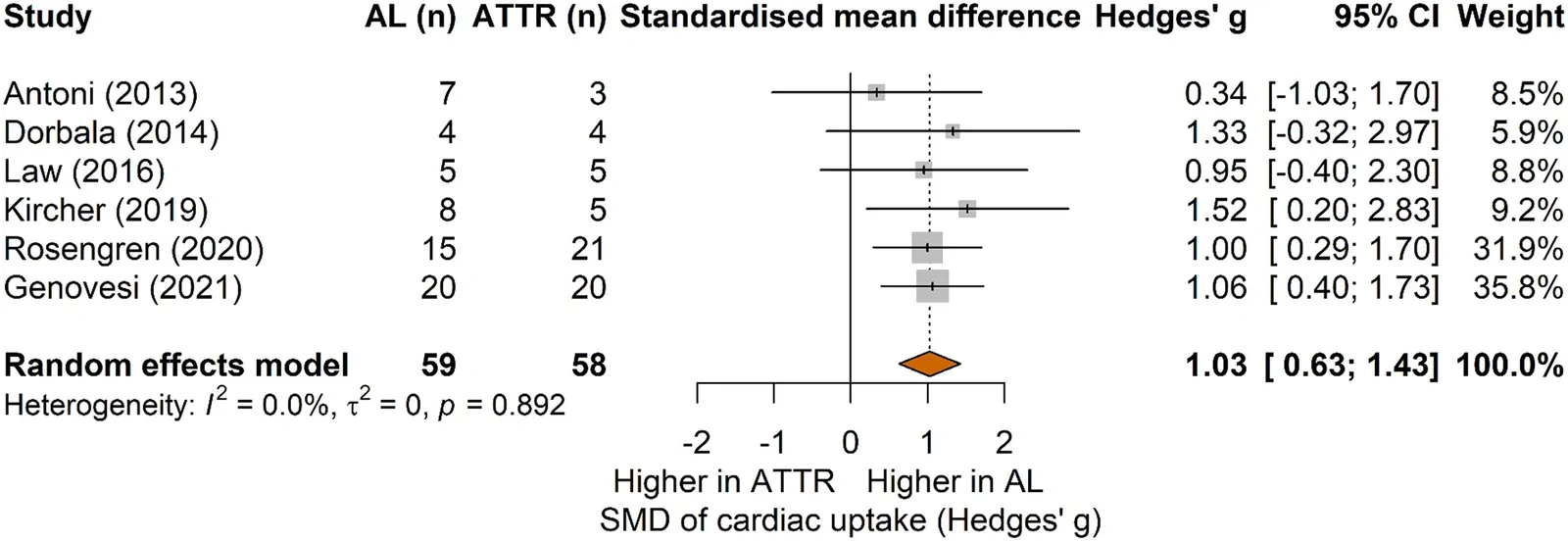
Quantitative myocardial uptake in AL vs. ATTR amyloidosis (standardized mean difference). Forest plot of the standardized mean difference (Hedges’ g) in quantitative myocardial uptake between AL and ATTR across six β-amyloid studies. Positive values indicate higher uptake in AL. Pooled by random-effects model (restricted maximum-likelihood). AL, light-chain amyloidosis; ATTR, transthyretin amyloidosis; CI, confidence interval; *I*^2^, heterogeneity statistic; SMD, standardized mean difference.

**Quantitative myocardial uptake** (AL vs. ATTR, *Figure 6*). Across the six β-amyloid studies reporting a quantitative retention metric by subtype, pooled myocardial uptake was higher in AL than in ATTR (standardized mean difference 1.03, 95% CI 0.63 to 1.43, *P* < 0.001), with no heterogeneity (*I*^2^ = 0%) and an entirely positive prediction interval (0.50 to 1.55). Restricting the analysis to retention-index studies yielded a near-identical estimate (SMD 0.98, *I*^2^ = 0%).

### Heterogeneity and publication bias

Between-study heterogeneity was low in both univariate models (sensitivity *I*^2^ = 13.4%; specificity *I*^2^ = 0.0%), below the pre-specified threshold for meta-regression (*I*^2^ > 50%), which was therefore not performed. Deeks’ funnel-plot asymmetry test was significant (*P* = 0.023; Supplementary data online, *Figure S3*), but interpretation is limited by the modest number of studies (*k* = 17, below the recommended ≥ 25) and by clustering at zero false-positive rates, which amplifies the continuity-correction influence in small samples.

## Discussion

This systematic review and meta-analysis synthesized data on the diagnostic accuracy of five amyloid-binding PET tracers in CA from 20 studies involving a total of 577 patients. In the primary bivariate model (*k* = 17), amyloid PET demonstrated high diagnostic accuracy with a pooled sensitivity of 0.91, a specificity of 0.90, and a SROC AUC of 0.96, with low statistical heterogeneity in diagnostic estimates (sensitivity *I*^2^ = 13.4%, specificity *I*^2^ = 0%). These findings support the diagnostic potential of amyloid PET as a non-invasive tool for CA, although the small number and size of the included studies warrant cautious interpretation of the pooled estimates.

### Comparison with literature

Our results are largely consistent with previous meta-analyses on the diagnostic accuracy of PET in CA, although reported estimates varied depending on study design. Two earlier meta-analyses^33,34^ reported very high accuracy. One^33^ pooled six studies and reported a sensitivity of 0.95 and specificity of 0.98, while a more comprehensive one^34^ assessed 13 studies and reported a sensitivity of 0.97 and a specificity of 0.98 for amyloid-binding tracers alone. In the latter, pooling amyloid tracers together with ^18^F-sodium fluoride, which targets microcalcification rather than amyloid, led to a significant increase in heterogeneity (*I*^2^ = 81%), although each tracer class alone was homogeneous. The near-perfect specificity (0.98) with no heterogeneity (*I*^2^ = 0%) in both these meta-analyses reflects a ceiling effect: when almost all studies report a specificity at or close to 1.00, between-study variance collapses and univariate pooling returns unrealistically perfect estimates. We observed the same phenomenon in our data, where specificity reached 1.00 in 16 of 17 studies on univariate pooling. We therefore used a bivariate (Reitsma) model as our primary analysis, which jointly models sensitivity and specificity and yields slightly lower but more realistic estimates (sensitivity 0.91; specificity 0.90). Other meta-analyses reported lower estimates, again reflecting study design rather than true tracer performance. A multimodality meta-analysis^35^ compared CMR, SPECT, and PET and reported a lower pooled PET sensitivity (0.78; specificity 0.95; AUC 0.95; *I*^2^ = 85% for sensitivity), as its pool combined amyloid-binding tracers with ^18^F-sodium fluoride and a single ^18^F-flutemetamol study (sensitivity 0.17). Their isolated ^11^C-PiB subgroup (sensitivity 0.91; specificity 0.97; AUC 0.98) closely matched our estimates. A qualitative systematic review^36^ focused specifically on ATTR cardiomyopathy and explicitly abstained from meta-analysis because of clinical heterogeneity in imaging protocols, outcomes, and control groups. A broad systematic review of PET across systemic amyloidosis^37^ reported, in a seven-study cardiac subgroup, a high sensitivity (0.98) but a low and highly heterogeneous specificity (0.61; *I*^2^ = 95%). This low specificity is driven by studies that lacked a non-amyloid control group, enrolled only AL patients, and used amyloid patients without cardiac involvement as the comparator,^13–15^ so that PET detection of early or subclinical cardiac amyloid was counted as a false positive. We excluded such studies and used exclusively amyloid-negative controls (healthy subjects or patients with heart disease without amyloid formation), so that our specificity (0.90) falls between this biased estimate and the near-ceiling values reported in earlier analyses.

### Performance by radiotracer

Beyond the pooled estimate, diagnostic performance varied by radiotracer. Sensitivity was high and consistent across ^11^C-PiB, ^18^F-florbetaben, ^18^F-florbetapir, and ^124^I-evuzamitide (0.96–1.00), with the exception of ^18^F-flutemetamol (0.70). The following sections discuss each tracer in turn.

^11^C-PiB was the most extensively studied tracer (*k* = 7) and showed the most consistent performance, with a pooled sensitivity of 0.96 (95% CI 0.88–0.99) and a specificity of 1.00 across the six controlled studies without heterogeneity (*I*^2^ = 0%). As in the earlier meta-analyses, this perfect specificity reflects a ceiling effect rather than perfect discrimination. Beyond the pooled estimate, two studies that did not contribute to the meta-analysis support and extend these findings. Pilebro *et al*.^38^ found ^11^C-PiB retention in all 10 ATTRv (V30M) patients, including ^99^ᵐTc-DPD-negative cases, with retention roughly three-fold higher in full-length than fragmented fibrils (0.129 vs. 0.040 min^−1^; *P* < 0.01), indicating that signal magnitude depends on fibril composition as well as amyloid burden. Beyond diagnosis, Choi *et al*.^39^ showed in 58 AL patients that ^11^C-PiB uptake independently predicted 1-year mortality (adjusted HR 3.38, 95% CI 1.01–11.32) after accounting for troponin, NT-proBNP, and dFLC. Two further studies^40,41^ combining ^11^C-PiB with a bone tracer improved AL-vs.-ATTR subtyping. Overall, ^11^C-PiB detects both subtypes with high accuracy and tends to show greater uptake in AL, but its short half-life limits use in routine clinical applications.

^18^F-florbetaben was assessed in four studies, with a pooled sensitivity of 0.99 (95% CI 0.49–1.00) and specificity of 0.99 (95% CI 0.05–1.00). Both intervals are wide given the small number of studies, and the very low lower bound of the specificity interval is driven by a single study. Its central feature is that detection and subtype discrimination depend on acquisition timing. In the largest florbetaben cohort to date^24^ (20 AL, 20 ATTR, 20 non-amyloid controls), early myocardial uptake was present in all groups, but signal was retained over time in AL and cleared rapidly in ATTR and non-amyloid hearts; myocardial washout between 5 and 60 min was 22% in AL vs. 74% in ATTR and 75% in non-amyloid hearts. Critically, on late and delayed images, ATTR uptake was statistically indistinguishable from non-amyloid hearts, so that florbetaben reliably identified AL but not ATTR against a non-amyloid background. Two studies^42,43^ that did not contribute to the meta-analysis clarify the mechanism: kinetic modelling showed irreversible tracer trapping in AL vs. reversible kinetics in ATTR and controls, and a neural-network classifier separated AL from ATTR on early images even when a blinded expert performed near chance. Beyond its cardiac application, whole-body florbetaben imaging has demonstrated extracardiac uptake across multiple organs and detected early multi-organ involvement before structural change.^44–47^ Overall, florbetaben detects AL-CA with high accuracy, whereas ATTR uptake was substantially lower.

^18^F-florbetapir was assessed in three studies, with pooled sensitivity and specificity both 1.00; however, only one provided non-amyloid controls, so the specificity estimate rests on a single study. Using ^18^F-florbetapir, myocardial uptake was found in both AL and ATTR, with higher uptake and stronger clinical correlations in patients with AL.^26^ Two studies that did not contribute to the meta-analysis temper this picture: Osborne *et al*.^48^ found that amyloid and control hearts separated only at later time points, not on early images, and Mestre-Torres *et al*.,^49^ using a single late whole-body acquisition rather than a cardiac-optimized early protocol, detected no cardiac uptake in any of three ATTR patients despite confirmed involvement. Florbetapir therefore detects cardiac amyloid but with time- and protocol-dependent performance in ATTR amyloidosis.

**^18^** F-flutemetamol showed the lowest and most heterogeneous pooled sensitivity of any tracer (0.70, 95% CI 0.21–0.95; *I*^2^ = 85.3%), with pooled specificity of 1.00. All three studies were ATTR-focused, so the heterogeneity reflects a wide range in performance rather than a subtype effect. Papathanasiou *et al*.^29^ found no significant difference in SUV or TBR between predominantly ATTR patients and non-amyloid controls, corresponding to a sensitivity of 0.17, whereas Möckelind *et al*.,^28^ in an exclusively variant-ATTR cohort, reached 0.88, although amyloid was confirmed only on fat-pad biopsy using an in-sample threshold. Papathanasiou *et al*. attributed the low sensitivity they observed to weak early myocardial kinetics dominated by perfusion, which in most patients did not exceed the background activity of the blood pool, and explicitly ruled out late imaging as an explanation, noting that earlier studies using comparably late time points had nevertheless achieved high diagnostic accuracy. However, Singh and Dorbala later argued that timing was in fact a key factor^50^: the markedly delayed image acquisition (≥59 min post-injection in 11 of 12 patients) fell well outside the 5–15-min window in which myocardial amyloid PET tracer activity maximally discriminates cardiac amyloidosis from controls, based on published time-activity curve data. They further noted that the predominantly ATTR-CM cohort (10 of 12 amyloidosis patients) and the comparatively low administered tracer activity (5 mCi) may have additionally contributed to the low sensitivity observed. This range indicates that additional research is needed on this tracer, consistent with prior reviews^36,51^ that likewise found the evidence on ^18^F-flutemetamol contradictory and limited by unharmonized protocols and thresholds.

Unlike the β-amyloid tracers, ^124^I-evuzamitide binds glycosaminoglycans, which are abundant on amyloid fibrils of any composition, making it pan-amyloid rather than β-sheet-selective. Across three studies, it achieved a pooled sensitivity of 0.99 (95% CI 0.91–1.00) and specificity of 1.00. A further study by Smiley *et al*.,^52^ not included in the meta-analysis owing to insufficient 2 × 2 data, identified myocardial amyloid in variant and wild-type ATTR subjects in whom ^99^ᵐTc-PYP was negative, indicating that ^124^I-evuzamitide has a very high affinity for ATTR amyloid deposits, making it particularly suitable for detecting the earliest phases of cardiac involvement.

### *AL* vs. *ATTR*

The previous tracer-specific findings also suggest a difference between the amyloid subtypes, which we examined on two levels: the likelihood of detection and the intensity of uptake. At the binary level, arm-level sensitivity was comparably high for both subtypes, although this pooled estimate combines all radiotracers. On a continuous scale, however, myocardial uptake across the six β-amyloid studies reporting a quantitative retention metric by subtype was higher in AL than in ATTR. This aligns with an earlier meta-analysis of semiquantitative PET parameters,^33^ which reported the same direction and magnitude based on fewer studies; restricting our analysis to the same retention-index metric reproduced their estimate almost exactly (SMD 0.98 vs. 0.96), supporting the robustness of this subtype difference. Beyond this difference in uptake intensity, a positive PET tended to be more frequent in AL than in ATTR in a previous meta-analysis,^37^ although with considerable between-study heterogeneity and a confidence interval crossing unity. This subtype difference is mechanistically plausible and applies specifically to the β-sheet–binding tracers, which showed higher myocardial uptake in AL than in ATTR. This difference remains incompletely explained given the likely greater amyloid load in ATTR myocardium.^51^ One contributing factor may be fibril composition, as ^11^C-PiB retention has been shown to depend on fibril type.^38^

### Limitations

Several limitations must be considered. First, this meta-analysis is based on a relatively small number of patients. Secondly, the included studies predominantly used case-control designs with separately recruited controls, often healthy volunteers or patients with left ventricular hypertrophy^22^ and hypertrophic cardiomyopathy,^20^ rather than consecutive patient cohorts with clinically suspected CA. This design tends to inflate diagnostic accuracy, so the reported estimates may overstate real-world performance in patients with suspected disease. Thirdly, imaging protocols and positivity thresholds varied considerably across studies, including scanners, tracer doses, imaging time points, evaluation methods, and quantitative cut-off values, some of which were derived through ROC optimization within the sample. Fourth, the reference standards varied across studies, ranging from endomyocardial biopsy to extracardiac biopsy combined with imaging criteria to consensus-based diagnostic algorithms. Standardized, prospectively applied reference criteria would strengthen future estimates of diagnostic accuracy. Fifth, several pooled estimates rest on a small number of studies and should be regarded as hypothesis-generating, particularly the tracer- and subgroup-specific results with wide confidence intervals. Lastly, Deeks’ funnel-plot asymmetry test was statistically significant, raising the possibility of publication bias or small-study effects. Its interpretation is, however, limited by the small number of contributing studies.

### Clinical implications and future directions

Bone scintigraphy already offers high diagnostic accuracy for ATTR-CA, provided that AL amyloidosis has been ruled out by serum and urine tests. For amyloid PET to serve as a non-invasive alternative in ATTR, it would need to achieve this level of accuracy. Two tracers stand out in this regard: ^18^F-florbetaben, which performs best in detecting AL, and ^124^I-evuzamitide, a pan-amyloid agent with high sensitivity for ATTR that may detect myocardial involvement at an early stage, including in cases with negative bone scintigraphy.

**Figure 7:**
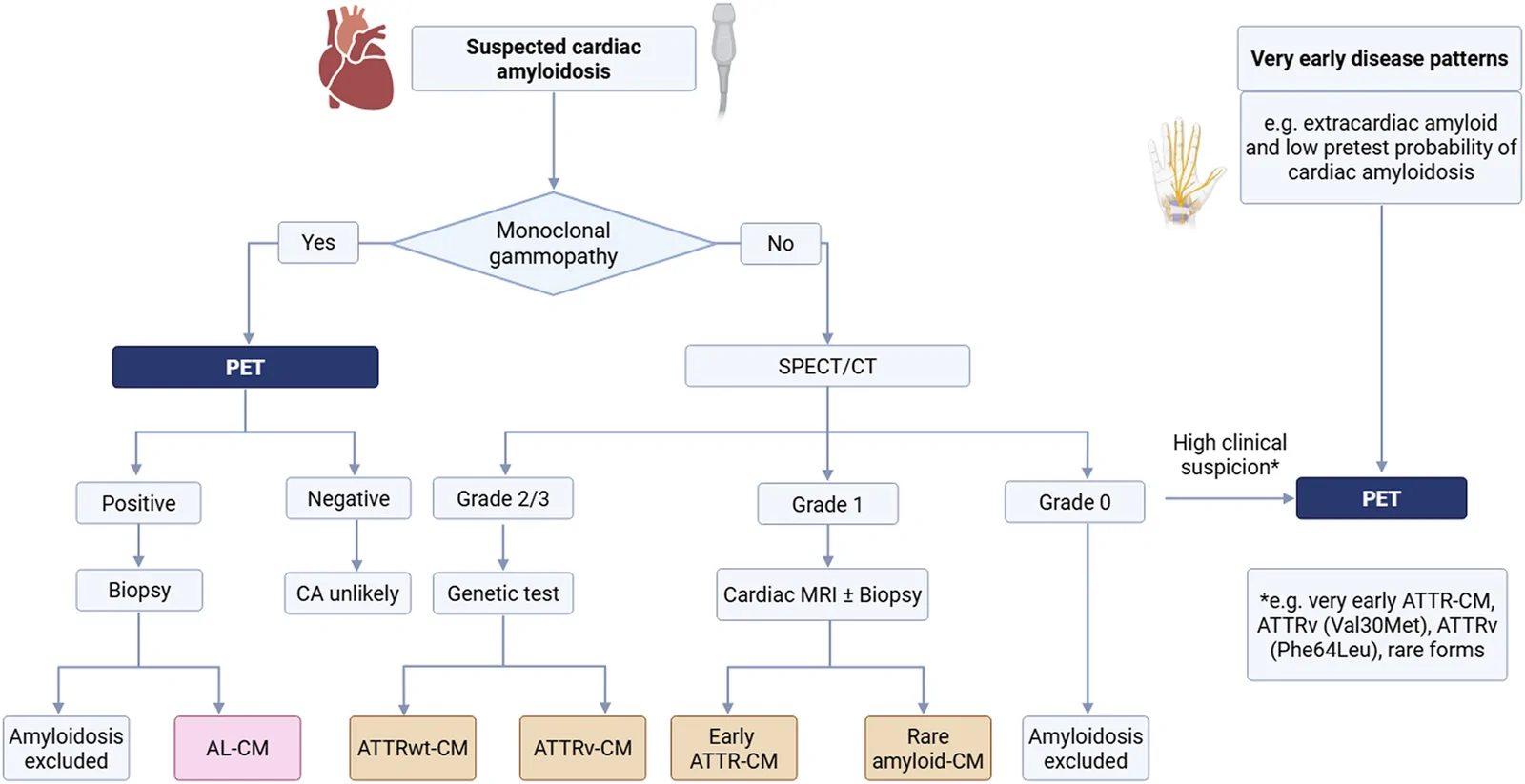
Potential future diagnostic workflow illustrating where amyloid PET may add value in the evaluation of suspected cardiac amyloidosis. AL-CM, immunoglobulin light-chain cardiac amyloidosis; ATTR-CM, transthyretin cardiac amyloidosis; ATTRv-CM, variant transthyretin cardiac amyloidosis; ATTRwt-CM, wild-type transthyretin cardiac amyloidosis; CA, cardiac amyloidosis; CM, cardiomyopathy; CT, computed tomography; MRI, magnetic resonance imaging; PET, positron emission tomography; SPECT, single-photon emission computed tomography. Created in BioRender. Walser, A. (2026).

Although amyloid PET has the potential to detect all forms of CA, its greatest clinical value may emerge in selected patient populations (*Figure 7*). These include patients with suspected CA and a monoclonal gammopathy, where demonstration of cardiac amyloid deposits could strengthen the indication for endomyocardial biopsy or guide biopsy towards extracardiac sites with demonstrable amyloid involvement. Amyloid PET may also prove valuable in patients with high clinical suspicion despite negative bone scintigraphy, particularly for ATTRv and rare amyloid subtypes that may be missed by conventional scintigraphic approaches. Furthermore, highly sensitive amyloid PET tracers may enable the identification of very early cardiac involvement in at-risk populations, such as patients with carpal tunnel syndrome and amyloid-positive surgical specimens but otherwise unremarkable cardiac evaluation, including normal electrocardiography, echocardiography, and cardiac biomarkers. This workflow represents a potential future application rather than an established clinical pathway; prospective validation in consecutive patients with suspected CA is required before implementation in routine practice.

An additional advantage of amyloid PET is its ability to visualize and quantify extracardiac amyloid deposits, which may contribute to disease staging and potentially assist in differentiating ATTR from AL amyloidosis based on the distribution of systemic amyloid burden. Beyond diagnosis, the quantitative nature of PET opens opportunities for risk stratification, assessment of cardiac amyloid burden, and serial monitoring of disease progression or treatment response—applications for which bone scintigraphy and current echocardiographic or CMR markers provide only indirect surrogates.^53^ These capabilities may become particularly important as amyloid-depleting therapies enter clinical practice, creating a need for robust biomarkers that can track changes in both cardiac and extracardiac amyloid burden over time. To realize this potential, harmonized imaging protocols, standardized quantification methods, and clearly defined positivity thresholds will be essential.

## Conclusion

Based on a still limited number of studies and patients, amyloid PET shows promising diagnostic accuracy for the detection of cardiac amyloidosis, and semiquantitative analysis may allow for the assessment of amyloid burden and differentiation of subtypes. Particularly with the pan-amyloid peptide tracer ^124^I-evuzamitide and the AL-specific agent ^18^F-florbetaben, amyloid PET offers the prospect of a non-invasive diagnosis across both AL and ATTR. Larger prospective studies in consecutive patients with suspected cardiac amyloidosis, using standardized protocols and positivity thresholds, are needed to determine diagnostic accuracy, particularly for ATTR.

## Supplementary Material

qyag141_Supplementary_Data

## Data Availability

All data underlying this review are available within the published studies included in the analysis.

## Lead author biography

Amely Walser is a PhD candidate at the University of Zurich and the University Hospital Zurich, Department of Nuclear Medicine, under the supervision of PD Dr Dominik Benz and Prof. Dr Philipp Kaufmann. Her research focuses on multimodal imaging of cardiac amyloidosis, encompassing nuclear imaging, echocardiography, and MRI.
