# Supplementary material for: Diagnostic value of positron emission tomography using amyloid-binding radiotracers for cardiac amyloidosis: a systematic review and meta-analysis
Source: Eur Heart J Imaging Methods Pract. 2026 Aug 18;4(3):qyag141. doi: 10.1093/ehjimp/qyag141 (PMC13537016; doi:10.1093/ehjimp/qyag141)
Supplement: Supplementary file 1 [file qyag141_supplementary_data.docx]

SUPPLEMENTARY MATERIAL

**Supplementary Figure S1**. **Risk of bias and applicability concerns of the 20 included studies according to QUADAS-2.**

*Alt text: Grid showing risk of bias and applicability concerns for 20 included studies according to QUADAS-2. Rows represent individual studies, columns represent the four risk-of-bias domains (patient selection, index test, reference standard, flow and timing) and three applicability domains. Each cell is colour-coded to indicate low, unclear, or high risk of bias or applicability concern.*

**Supplementary Figure S2. Forest plot of the diagnostic odds ratio (DOR) of amyloid-binding PET for the diagnosis of cardiac amyloidosis.**

*Grey squares represent study-specific DORs with areas proportional to study weight, horizontal lines the 95% confidence intervals, and the diamond the random-effects pooled estimate. A continuity correction of 0.5 was applied to cells with zero counts. Because 16 of 17 studies reported no false-positive results, the study-specific DORs and their confidence intervals are correspondingly large, and the pooled DOR is therefore driven primarily by sensitivity. Between-study heterogeneity was negligible (I² = 0%, p = 0.461).*

*CA+, patients with cardiac amyloidosis (reference-standard positive). CA−, non-amyloid controls. PET+, positive amyloid PET result. n, number of patients in the respective group.*

*Alt text: Forest plot of the diagnostic odds ratio of amyloid-binding PET for the diagnosis of cardiac amyloidosis. Grey squares represent study-specific estimates with areas proportional to study weight, horizontal lines indicate 95% confidence intervals, and a diamond represents the random-effects pooled estimate. Study-specific DORs are large due to the absence of false positives in most studies. Between-study heterogeneity was negligible.*

**Supplementary Figure S3.** **Deeks' funnel plot publication bias.**

*Deeks' funnel-plot asymmetry test (k = 17), regression slope significant (p = 0.023). Interpretation is limited by k < 25 and by clustering at zero false-positive rates. DOR, diagnostic odds ratio; ESS, effective sample size.*

*Alt text: Deeks' funnel plot assessing publication bias across 17 studies. Diagnostic odds ratio is plotted against effective sample size. A regression line is shown, with statistically significant asymmetry (p = 0.023). Interpretation is limited by the small number of studies and clustering at zero false-positive rates.*

**Supplementary Table S1. Full Search Strategy**

| Database | Search string |
| --- | --- |
| PubMed (MEDLINE) | ("cardiac amyloidosis" OR "amyloid cardiomyopathy" OR "AL amyloidosis" OR "ATTR amyloidosis" OR "systemic amyloidosis") AND ("positron emission tomography" OR "PET" OR "florbetapir" OR "florbetaben" OR "flutemetamol" OR "evuzamitide" OR "Pittsburgh compound B" OR "PIB" OR "PiB" OR "C-11 PIB") |
| Embase | ('cardiac amyloidosis':ti,ab OR 'amyloid cardiomyopathy':ti,ab OR 'al amyloidosis':ti,ab OR 'attr amyloidosis':ti,ab OR 'systemic amyloidosis':ti,ab) AND ('positron emission tomography':ti,ab OR 'pet':ti,ab OR 'florbetapir':ti,ab OR 'florbetaben':ti,ab OR 'flutemetamol':ti,ab OR 'evuzamitide':ti,ab OR 'pittsburgh compound b':ti,ab OR 'pib':ti,ab OR 'c-11 pib':ti,ab) |
| Cochrane Library | ("cardiac amyloidosis" OR "amyloid cardiomyopathy" OR "AL amyloidosis" OR "ATTR amyloidosis" OR "systemic amyloidosis") AND ("positron emission tomography" OR "PET" OR "florbetapir" OR "florbetaben" OR "flutemetamol" OR "evuzamitide" OR "Pittsburgh compound B" OR "PIB" OR "PiB" OR "C-11 PIB") |
| Web of Science | TS=(("cardiac amyloidosis" OR "amyloid cardiomyopathy" OR "AL amyloidosis" OR "ATTR amyloidosis" OR "systemic amyloidosis") AND ("positron emission tomography" OR "PET" OR "florbetapir" OR "florbetaben" OR "flutemetamol" OR "evuzamitide" OR "Pittsburgh compound B" OR "PIB" OR "PiB" OR "C-11 PIB")) |

**Supplementary Table S2. Data Extraction Items**

| Category | Data items extracted |
| --- | --- |
| Study identification | Author (first author)  Year of publication  DOI / PMID  Country of origin  Single-center vs multi-center |
| Study design | Prospective vs retrospective  Inclusion period (start / end dates)  Consecutive vs case-control recruitment |
| Patient characteristics | Total number of patients  Number of cardiac amyloidosis patients  Number of controls  Amyloid subtype (AL / ATTR / Mixed / Unknown)  ATTR subtype where applicable (wild-type vs variant)  Control group composition (healthy controls, disease controls, AL non-CA, mixed)  Mean or median age (years), with range where reported  Sex distribution (% male) |
| Index test characteristics | Radiotracer used (¹¹C-PiB, ¹⁸F-florbetaben, ¹⁸F-florbetapir, ¹⁸F-flutemetamol, ¹²⁴I-evuzamitide)  PET modality (PET/CT vs PET/MR)  Tracer dose (MBq or MBq/kg)  Dynamic scan duration (min post-injection)  Static scan window (start–duration, min post-injection)  Reading method (visual / quantitative / both)  Quantitative parameters reported (e.g., SUVmax, SUVmean, SUVR, TBR, retention index)  Cut-off value used to classify a positive scan |
| Reference standard | Primary reference standard (endomyocardial biopsy, extra-cardiac biopsy, scintigraphy + biomarker, etc.)  Diagnostic criteria applied (Gertz criteria, ISA consensus, etc.)  Time interval between index test and reference standard, where reported |
| Diagnostic accuracy outcomes | Sensitivity with 95% confidence interval (Clopper-Pearson)  Specificity with 95% confidence interval (Clopper-Pearson)  Area under the curve (AUC), if reported  2×2 contingency table: true positives, false positives, false negatives, true negatives |
| Methodological quality | QUADAS-2 ratings across four risk-of-bias domains: patient selection, index test, reference standard, flow and timing  QUADAS-2 ratings across three applicability domains: patient selection, index test, reference standard |
| Quantitative myocardial uptake (AL versus ATTR subgroup analysis) | SMD analysis eligibility, with reason for exclusion where not eligible  Chosen retention metric (retention index preferred)  Reporting format (mean ± SD or median with IQR/range)  By-subtype values for AL and ATTR: mean ± SD or median (IQR/range), and n per subtype arm |

**Supplementary Table S3. Cohort Overlap Assessment**

To prevent double-counting of patients in the quantitative synthesis, all studies retrieved at full-text screening were systematically assessed for potential overlap in study populations. Overlap was suspected when two or more studies shared the same research group or institutional collaboration, overlapping recruitment periods, similar imaging protocols and tracer selection, or comparable patient demographics in published cohort summaries. Where overlap was confirmed or strongly suspected, the publication with the largest sample size, most complete reporting, or most rigorous design was retained for quantitative synthesis. Three studies were excluded on this basis (see table below).

Studies excluded from quantitative synthesis due to cohort overlap.

| Excluded study | Retained study | Rationale |
| --- | --- | --- |
| Zhou (2023) | Wang (2022)^15^ | Both from Wuhan Union Hospital research group; overlapping AL cohort (Jan 2019–Oct 2021). Wang retained as peer-reviewed full text with complete 2×2 data. |
| Cassano (2024)^53^ | Genovesi (2021)^26^ | Both from Pisa registry (Fondazione Toscana G. Monasterio + Scuola Sant'Anna). Genovesi retained as larger, methodologically more complete cohort with full dynamic PET acquisition. |
| Giorgetti (2026)^54^ | Genovesi (2021)^26^ | Continuation of the Pisa registry. Genovesi retained per same rationale; Giorgetti reported a machine-learning reanalysis not relevant to the present diagnostic-accuracy synthesis. |

For two pairs that may share a single-centre cohort (Ezawa^45^ and Takasone^24^; Genovesi^26^ and Santarelli^28^), only one study of each pair contributed to the quantitative synthesis, as the other reported a combined-modality or non-standard analysis without an extractable 2×2 table, so the pooled estimates are unaffected by any patient-level overlap.

The table lists only studies excluded because of patient overlap with a separately published, independently retained study. Other records excluded at this stage were conference abstracts or preliminary reports later superseded by a full-text publication of the same cohort, and are not listed individually.
